# Circulating small non coding RNA signature in head and neck squamous cell carcinoma

**DOI:** 10.18632/oncotarget.4266

**Published:** 2015-05-25

**Authors:** Berta Victoria Martinez, Joseph M. Dhahbi, Yury O. Nunez Lopez, Katarzyna Lamperska, Paweł Golusinski, Lukasz Luczewski, Tomasz Kolenda, Hani Atamna, Stephen R. Spindler, Wojciech Golusinski, Michal M. Masternak

**Affiliations:** ^1^ University of Central Florida, Burnett School of Biomedical Sciences, College of Medicine Orlando, FL, USA; ^2^ Department of Biochemistry, University of California at Riverside, Riverside, CA, USA; ^3^ Translational Research Institute for Metabolism and Diabetes, Florida Hospital, Orlando, FL, USA; ^4^ Deptartment of Cancer Genetics, Greater Poland Cancer Centre, Poznan, Poland; ^5^ Department of Biology and Environmental Studies, Poznan University of Medical Sciences, Poznan, Poland; ^6^ Department of Head and Neck Surgery, Greater Poland Cancer Centre, Poznan University of Medical Sciences, Poznan, Poland; ^7^ Postgraduate School of Molecular Medicine, Medical University of Warsaw, Warszawa, Poland; ^8^ Department of Medical Education, California Northstate University, Elk Grove, CA, USA

**Keywords:** head and neck cancer, next-generation sequencing, circulating small RNAs, microRNAs, tRNA halves

## Abstract

The Head and Neck Squamous Cell Carcinoma (HNSCC) is the sixth most common human cancer, causing 350,000 individuals die worldwide each year. The overall prognosis in HNSCC patients has not significantly changed for the last decade. Complete understanding of the molecular mechanisms in HNSCC carcinogenesis could allow an earlier diagnosis and the use of more specific and effective therapies. In the present study we used deep sequencing to characterize small non-coding RNAs (sncRNAs) in serum from HNSCC patients and healthy donors. We identified, for the first time, a multi-marker signature of 3 major classes of circulating sncRNAs in HNSCC, revealing the presence of circulating novel and known miRNAs, and tRNA- and YRNA-derived small RNAs that were significantly deregulated in the sera of HNSCC patients compared to healthy controls. By implementing a triple-filtering approach we identified a subset of highly biologically relevant miRNA-mRNA interactions and we demonstrated that the same genes/pathways affected by somatic mutations in cancer are affected by changes in the abundance of miRNAs. Therefore, one important conclusion from our work is that during cancer development, there seems to be a convergence of oncogenic processes driven by somatic mutations and/or miRNA regulation affecting key cellular pathways.

## INTRODUCTION

Head and Neck Squamous Cell Carcinoma (HNSCC) is the sixth most common human cancer. Close to 600,000 new patients are diagnosed and approximately 350,000 individuals die of this disease worldwide each year [1, 2]. Tobacco use and alcohol consumption are the major risk factors for HNSCC [3]. Lastly, infection with high-risk types of human papillomavirus (HPV) has been identified as a novel risk factor for a subset of HNSCCs, particularly those of the oropharynx [4]. Recent advances in HNSCC treatment have improved the quality of life and life expectancy of HNSCC patients if this disease is diagnosed at early stages [5]. However, the overall prognosis in HNSCC patients has not significantly changed for the last decade [6]. Currently, the most common HNSCC therapeutic modalities include the use of nonselective treatments (surgery, radiation and chemotherapy) with very high systemic toxicities and associated morbidity and mortality. Complete understanding of the molecular mechanisms in HNSCC carcinogenesis (identifying actionable targets of therapeutic value) will benefit the development of more selective cancer treatment options for HNSCC. Head and neck carcinogenesis is a multistep process driven by an accumulation of several genetic and epigenetic alterations in oncogenes and tumor suppressor genes leading to the progression of a normal cell to a cancer cell [7]. HNSCC cells are genetically unstable and often display extensive chromosomal changes, including amplifications, deletions, and translocations. Recently, next-generation sequencing (NGS) studies have revealed that the genetic alterations in HNSCC are mainly in a group of molecular pathways and/or biological processes including p53 pathways (TP53), mitogenic pathways (RAS/PI3K/mTOR pathway, PIK3CA, HRAS), cell cycle (CDKN2A), Notch pathways (NOTCH1, NOTCH2, NOTCH3), and cell communication and death (FAT1, CASP8) [8] (Reviewed in [9]).

On the other hand, advanced cancer studies suggest small non-coding RNAs (sncRNAs) as useful biomarkers of cancer development and determination of tumor stage. MicroRNAs are a type of sncRNA that regulate diverse biological processes. Each miRNA can control hundreds of gene targets, underscoring the potential influence of miRNAs on almost every genetic pathway. Recent evidence has shown that miRNA mutations or mis-expression correlate with various human cancers and indicates that miRNAs can function as tumor suppressors and/or oncogenes (Reviewed in [10]). There is increasing number of studies on miRNAs expression in HNSCC (mainly in tumor tissue samples). However, once the patients undergo surgical intervention and tumor tissue is removed, it is difficult to determine the risk of disease recurrence or death. The presence of sncRNAs circulating in the blood provides a new venue for easy access to non-invasive frequent screening of patients.

The recent advent of NGS expedited the identification of new types of small RNAs; specifically, known sncRNAs including tRNA, rRNA, and YRNA can undergo processing into smaller RNA molecules [11-13] that can function in normal biology and in pathologic conditions [13-19]. In addition to miRNAs, our deep sequencing studies of serum/plasma have consistently detected two other major classes of extracellular small RNAs: tRNA-derived RNAs of size 30-33 nt, and YRNA-derived RNAs of sizes 27 nt and 30-33 nt [20, 21]. These new sncRNAs have only recently emerged because early studies focused on miRNAs and systematically excluded sequencing reads whose length exceeded the size of mature miRNAs (18-24 nt) or reads that align with tRNAs and rRNAs since they were considered degradation products. Similarly to miRNAs, these derivatives of sncRNAs can be released into the extracellular environment and thereby carry paracrine and even endocrine signaling functions [22]. Accumulating evidence suggests they may function in cell-to-cell communication both in normal biology and in disease states [23-26]. We have found that aging changes the circulating levels of 5′ tRNA halves derived from specific tRNA isoacceptors and the age-induced changes can be mitigated by calorie restriction [21]. This alleviation of the age-associated changes by calorie restriction reveals functional significance because it validates the age-associated alterations in the levels of circulating 5′ tRNA halves, and provides further evidence that circulating 5′ tRNA halves are physiologically regulated. More recently, we have reported that breast cancer and its clinicopathological characteristics can be associated with changes in serum levels of specific types of both YRNA- and tRNA-derived small RNAs [27]. The presence of these new small RNAs in serum/plasma, as well as their close associations with relevant pathophysiological processes such as aging and cancer, should spark strong interest in developing them into noninvasive diagnostic biomarkers and therapeutic targets.

In the present study using NGS, we characterized for the first time three major classes of circulating sncRNAs (miRNA, tRNA- and YRNA-derived small RNAs) in serum from HNSCC patients and healthy donors. Our analysis revealed the presence of circulating novel and known miRNAs, 5′ tRNA halves, and 5′ or 3′ YRNA-derived small RNAs that were significantly deregulated in the HNSCC patients as compared to the healthy controls.

## RESULTS

### Characterization of small RNAs circulating in serum of normal subjects and oral cancer patients

**Figure 1:**
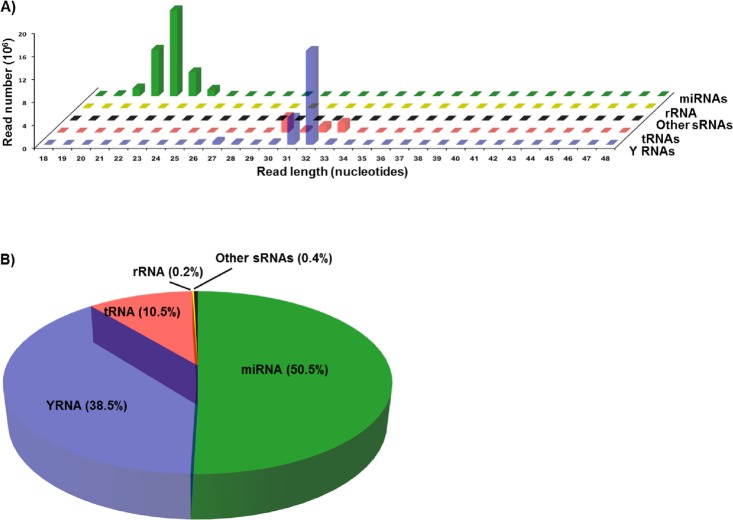
Length distribution and annotation of sequencing reads from serum small RNAs Sequencing reads from both normal and cancer samples were pooled to analyze the quality of reads, i.e., length distribution, and the types and proportions of small RNAs from which the reads are derived from. Pooling is used only to examine the general characteristics of the reads, and not to measure the differential expression of small RNAs between control and cancer groups. **A.** Plot of length against abundance of pooled mapped reads according to their annotation as miRNAs, YRNAs, tRNAs, rRNAs, or other sRNAs (snRNAs and snoRNAs). **B.** Pie chart showing the percent of reads mapping to the indicated types of small RNAs in pooled datasets obtained by sequencing of small RNAs in the sera of normal and cancer cases.

We used deep sequencing to investigate the changes in circulating small RNAs associated with oral cancer. The sequenced small RNAs were extracted from the sera of 7 male patients with oral cancer (Table 1) and 7 adult males with no known pathologies at the time of blood collection. Sequencing reads from both normal and cancer samples were pooled to determine the general characteristics of the reads, i.e., length distribution of the reads, the pattern and size of peaks, and the types and proportions of small RNAs from which the reads are derived from. Pooling is used only to examine the characteristics of the reads, and not to measure the differential expression of small RNAs between control and cancer groups. A combined total of 79,944,976 pre-processed reads were aligned to the hg19 human genome to generate a dataset of 67,401,008 mapped reads (84%), ranging in size from 18 to 48 nt. Annotation and length distribution analyses revealed that reads in the 20-24 nt peak were derived from miRNAs, while the 30-33 nt peak consists of reads mapping to both YRNA and tRNA genes (Figure 1A). Annotation analysis showed that of the total reads mapping to known small RNAs, 50% were annotated as miRNAs, 38% as YRNAs, and 10% as tRNAs (Figure 1B). The remaining < 1% of reads mapped to sequences annotated as rRNA, snRNA and snoRNA. These results are consistent with our previous findings [20, 21]. Interestingly, we detected an extremely statistically significant difference in the distribution of small RNA reads in the HNSCC group as compared to the normal group (Chi-square *P* < 0.0001, Supplementary Figure S1 and S2). The proportion of miRNA reads significantly increased in HNSCC patients and accounted for 66.4% of total reads as compared to 39.6% in the normal group. Correspondingly, HNSCC tRNAs and YRNAs dramatically decreased their proportions and accounted for 3% and 30.2% respectively, as compared to 15.6% and 44.2% in normal subjects. This suggests a remodeling of the small non-coding RNA networks in HNSCC. We did not find age as a determining factor in the observed changes in the levels of small RNAs as evidence by the lack of significant correlation between the subjects' age and the normalized expression levels of differentially abundant small RNAs.

**Table 1:** HNSCC cancer patients and healthy controls data

	Age	TNM	Tumor localization
**HNSCC patients**			
T1	57	T4N2M0	Oropharynx
T2	46	T3N1M0	Oropharynx
T3	61	T3N2M0	Oropharynx
T4	63	T3N1M0	Oropharynx
T5	58	T2N1M0	Oral
T6	63	T4N1M0	Oropharynx and Larynx
T7	46	T4N0M0	Oral and Oropharynx
**Healthy subjects**			
N1	64	N/A	N/A
N2	64	N/A	N/A
N3	66	N/A	N/A
N4	68	N/A	N/A
N5	69	N/A	N/A
N6	61	N/A	N/A
N7	66	N/A	N/A

TNM: Tumor extent (T), node status (N) and distant metastasis (M) categories

### Multi-dimensional scaling analysis

**Figure 2:**
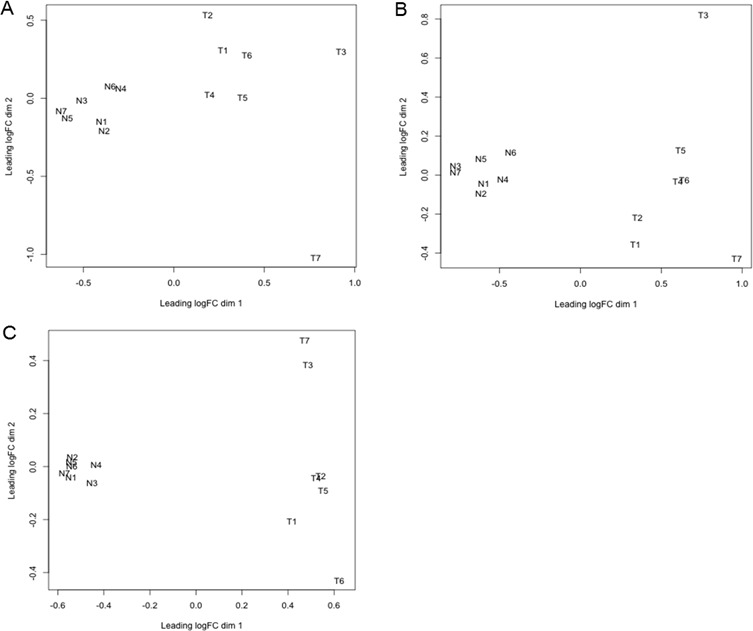
Multi-dimensional scaling (MDS) plot of circulating small RNAs. The plotMDS function of edgeR was used to examine relationship between samples of circulating miRNA **A.,** tRNA-derived small RNA **B.**, and YRNA-derived small RNA **C.**. This function clusters the small RNA samples according to two automatically determined dimensions (dim 1 and 2). Dimension 1 represents the cancer effect, while dimension 2 represents the homogeneity between biological replicates. The analyzed small RNA samples are from normal controls (N1-7), and cancer patients (T1-7). “logFC” is log fold change.

Before proceeding to the statistical analysis of the differential abundance (DA) of circulating small RNAs between normal and cancer cases, we used the plotMDS function of edgeR to examine the samples quality. The multi-dimensional scaling function displays pairwise similarity of each sample in two automatically determined dimensions; the plot separates the samples according to the expression levels and homogeneity of the replicates. The analysis shows clear separation between tumor and normal conditions, revealing distinct effects of cancer on the abundance of all 3 types of circulating small RNAs (Figure 2A-2C). However, the homogeneity of the replicates is more marked in the normal than in the tumor samples.

### Analysis of differential expression of circulating small RNAs between normal subjects and oral cancer patients

#### miRNAs

**Figure 3:**
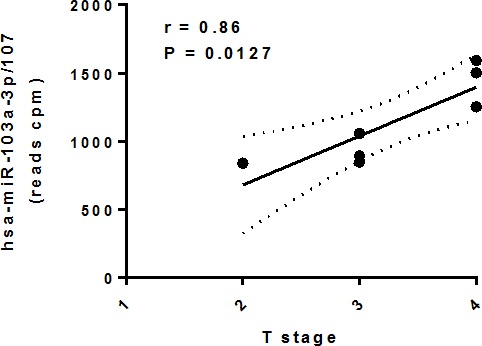
Association between differentially abundant circulating miR-103a-3p/miR-107 and HNSCC T stage Circulating miRNA levels were tested for normal distribution using the Shapiro-Wilk Normality Test and subjected to correlation analysis (r: Pearson correlation) in GraphPad Prism 6.05.

To measure the DA of circulating miRNAs between normal subjects and cancer patients, the sequencing reads from each serum sample were pre-processed and analyzed with miRDeep2 [28], which detects known and novel miRNAs and reports their expression levels. Our study revealed significant differences in the levels of 7 novel (*P* < 0.05, FDR < 0.10) and 28 known (*P* < 0.05, FDR < 0.15) miRNAs in serum from HNSCC patients as compare with healthy donors. Among the novel DA miRNAs, 3 were significantly downregulated while 4 were significantly upregulated (Table 2). Among the known DA miRNAs, 13 were significantly upregulated and 15 were significantly downregulated in serum from HNSCC patients as compared to healthy controls (Table 2). Importantly, upregulated circulating miR-103a-3p/107 demonstrated significant positive correlation with the size and/or extent (reach) of the primary tumor (*r* = 0.86, *P* = 0.0127, Figure 3).

**Table 2:** Novel and known circulating miRNAs differentially regulated by HNSCC

Provisional ID^1^	Genomic coordinates^3^	miRDeep2 score^4^	Estimated probability^5^	CPM^6^	FC^7^	Pvalue^7^	FDR^7^
chr3_6283	chr3:53118889..53118935:+	1.5	0.37 ± 0.04	**7**	**6.8**	1.19E-03	1.15E-02
chr17_28999	chr17:75803646..75803705:+	1.8	0.37 ± 0.04	**7**	**5.5**	9.79E-04	9.68E-03
chr6_12928	chr6:11631870..11631921:-	1.6	0.37 ± 0.04	**7**	**4.5**	2.63E-03	2.06E-02
chr8_16791	chr8:56821958..56822010:-	1.9	0.37 ± 0.04	**7**	**4.2**	5.35E-03	3.62E-02
chr7_15527	chr7:142157355..142157408:-	4.8	0.77 ± 0.06	**8**	**−2.8**	3.37E-04	4.94E-03
chr16_28195	chr16:58762523..58762572:-	4.0	0.77 ± 0.06	**59**	**−2.8**	3.03E-09	2.96E-07
chr9_17997	chr9:133282130..133282175:+	1.6	0.37 ± 0.04	**1092**	**−3.1**	1.92E-04	3.00E-03
**miRNA ID^2^**						
hsa-mi R-205-5p	chr1:209605511..209605569:+	460.0	0.87 ± 0.07	16	4.3	3.22E-04	4.81E-03
hsa-miR-145-5p	chr5:148810224..148810285:+	620.0	0.87 ± 0.07	13	2.2	5.81E-04	6.70E-03
hsa-miR-27b-5p	chr9:97847745..97847807:+	40000.0	0.87 ± 0.07	931	2.2	8.05E-05	1.65E-03
hsa-miR-103a-3p	chr5:167987912..167987968:-	4.7	0.77 ± 0.06	856	2.0	2.24E-03	1.81E-02
hsa-miR-107	chr10:91352516..91352572:-	4.7	0.77 ± 0.06	856	2.0	2.24E-03	1.81E-02
hsa-miR-320a	chr8:22102489..22102539:-	5.6	0.89 ± 0.05	225	1.9	5.48E-04	6.61E-03
hsa-miR-320b	chr1:224444752..224444801:-	5.0	0.89 ± 0.05	225	1.9	5.48E-04	6.61E-03
hsa-miR-486-5p	chr8:41517962..41518025:+	8000000.0	0.87 ± 0.07	258635	1.9	5.01E-05	1.16E-03
hsa-miR-100-5p	chr11:122022947..122023004:-	4.7	0.77 ± 0.06	185	1.9	1.39E-02	7.14E-02
hsa-miR-32-5p	chr9:111808511..111808573:-	5.4	0.89 ± 0.05	26	1.9	3.05E-02	1.20E-01
hsa-miR-215-5p	chr1:220291218..220291278:-	4.4	0.77 ± 0.06	33	1.8	2.76E-02	1.13E-01
hsa-miR-148a-5p	chr7:25989541..25989601:-	38000.0	0.87 ± 0.07	1310	1.8	1.54E-04	2.50E-03
hsa-miR-99a-5p	chr21:17911421..17911481:+	5.4	0.89 ± 0.05	66	1.8	4.33E-02	1.49E-01
hsa-miR-191-5p	chr3:49058063..49058127:-	150000.0	0.87 ± 0.07	4948	−2.2	2.15E-08	1.89E-06
hsa-miR-26a-5p	chr3:38010904..38010965:+	95000.0	0.87 ± 0.07	3131	−2.4	1.01E-05	3.57E-04
hsa-miR-181a-5p	chr1:198828197..198828259:-	84000.0	0.87 ± 0.07	2673	−1.8	6.19E-05	1.33E-03
hsa-miR-150-5p	chr19:50004055..50004110:-	60000.0	0.87 ± 0.07	1839	−6.1	5.55E-08	4.44E-06
hsa-let-7f-5p	chr9:96938635..96938713:+	20000.0	0.87 ± 0.07	725	−2.2	6.34E-04	6.88E-03
hsa-miR-93-5p	chr7:99691398..99691460:-	23000.0	0.87 ± 0.07	712	−1.9	1.94E-03	1.66E-02
hsa-let-7a-5p	chr22:46508632..46508702:+	14000.0	0.87 ± 0.07	535	−2.1	1.11E-04	2.06E-03
hsa-miR-30c-5p	chr6:72086668..72086728:-	10000.0	0.87 ± 0.07	353	−3.4	9.28E-07	4.30E-05
hsa-miR-28-5p	chr3:188406582..188406644:+	19000.0	0.87 ± 0.07	352	−1.8	5.15E-04	6.57E-03
hsa-miR-26b-5p	chr2:219267380..219267436:+	7200.0	0.87 ± 0.07	188	−2.1	2.02E-07	1.37E-05
hsa-miR-30b-5p	chr8:135812774..135812834:-	2600.0	0.87 ± 0.07	86	−5.3	1.82E-14	5.34E-12
hsa-miR-122-5p	chr18:56118320..56118377:+	760.0	0.87 ± 0.07	25	−3.9	9.65E-04	9.65E-03
hsa-miR-98-5p	chrX:53583201..53583281:-	770.0	0.87 ± 0.07	20	−2.7	1.39E-05	4.40E-04
hsa-miR-183-5p	chr7:129414767..129414827:-	1100.0	0.87 ± 0.07	20	−2.1	4.15E-02	1.46E-01
hsa-miR-224-5p	chrX:151127056..151127123:-	480.0	0.87 ± 0.07	16	−3.3	1.39E-03	1.30E-02

1 Unique identification containing the chromosome and an arbitrary number assigned to the hairpin predicted by miRDeep2, and have an randfold p-value < 0.05.

2 Names of mature miRNAs of Homo sapiens in miRbase v.20 (GRCh37.p5) that match precursor sequences predicted by miRDeep2, and have an randfold p-value < 0.05.

3 Location of the miRNA precursor in the human hg19 genome.

4 The miRDeep2 score represents the log-odds probability of a sequence being genuine miRNA precursor versus the probability that it is a background hairpin.

5 The estimated probability that the miRNA candidate is a true positive.

6 Average known miRNA read counts-per-million computed over all libraries and taking into account the estimated dispersions and the libraries sizes. It represents a measure of the overall expression level of the miRNA

7 Fold change, Pvalue and FDR for differential abundance were computed by EdgeR from pairwise comparisons for each miRNA between the control and cancer groups.

**Figure 4:**
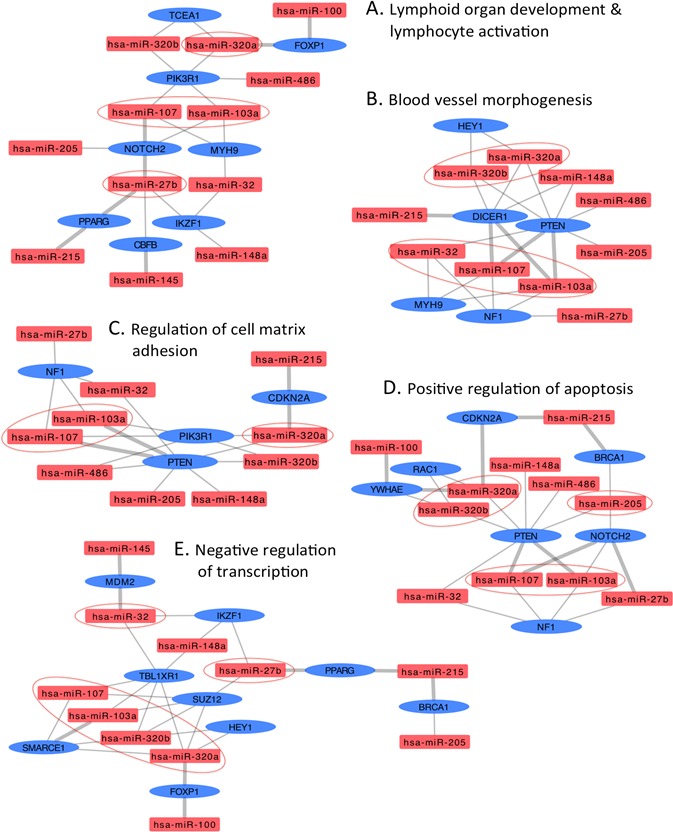
Interaction networks of COSMIC cancer consensus genes overtargeted by upregulated circulating HNSCC miRNAs Subnetworks represent functional categories significantly enriched in sets of genes overtargeted by upregulated miRNAs. **A.** lymphoid organ development & lymphocyte activation, **B.** blood vessel morphogenesis **C.** regulation of cell matrix adhesion, **D.** positive regulation of apoptosis, and **E.** negative regulation of transcription. Thick grey edges highlight validated miRNA-mRNA interactions, thin edges represent predicted interactions, ovals represent COSMIC genes overtargeted by upregulated HNSCC miRNAs (rectangles); color-coded red indicates upregulation, blue indicates downregulation. Circled miRNAs putatively target three or more genes in the specific subnetwork.

To determine the functional relevance of the DA miRNAs, we identified putative targets for each miRNA as described in the Materials and Methods section. Because prediction software identify a large number of putative miRNA targets that are not all biologically relevant, we implemented a triple-filtering approach that included: first, the over-representation analysis of all deregulated HNSCC miRNA targeting events on each predicted and validated target; second, the selection of overtargeted genes found to contain somatic mutations in cancer tissues (as reported by the COSMIC database); and third, the functional enrichment for KEGG pathways and GO annotations. This triple-filtering approach (accounting for miRNA overtargeting, somatic mutations in cancer, and pathway/GO category enrichment) allowed us to identify a relatively small subset of highly biologically relevant miRNA-mRNA interactions including 48 COSMIC genes overtargeted by upregulated miRNAs and 76 COSMIC genes overtargeted by downregulated miRNAs (Supplementary Table S1). The functional annotation analysis performed on this overtargeted COSMIC gene set identified several clusters of cancer-related biological processes such as regulation of apoptosis, cell cycle, blood vessel morphogenesis, immune system activation, and transcription among others that are key to development of HNSCC (Supplementary Tables S2 and S3). In Figures 3 and 4, we highlight these important HNSCC miRNA-mRNA interaction subnetworks. Genes overtargeted by miRNAs that are upregulated in the circulation of cancer patients are expected to be downregulated in the respective target tissues. On the contrary, genes overtargeted by miRNAs that are downregulated in the circulation of cancer patients are expected to be upregulated in the respective target tissues.

#### 5′ tRNA halves

We compared serum tRNA-derived small RNAs in datasets from HNSCC and normal subjects using the Bioconductor package edgeR [29] and found significant differential abundance of 5′ tRNA halves derived from a small number of tRNA genes; there are 625 human tRNA genes, each of them potentially capable of producing small RNAs. Our findings suggest that an HNSCC diagnosis is associated with alterations, either increase or decrease, in the circulating levels of 5′ tRNA halves derived from specific tRNA isoacceptors (Table 3). Individuals with HNSCC had significantly increased circulating levels of 5′ tRNA halves derived from isoacceptors of tRNA-Ala, -Cys, and -Tyr, and decreased circulating levels of 5′ tRNA halves derived from tRNA-Arg, -Glu, -Gly, -Lys, -Trp, and -Val.

**Table 3:** HNSCC-associated changes in the serum levels of 5′ tRNA halves

tRNA^1^	Genomic coordinates	CPM^2^	FC^3^	Pvalue^3^	FDR^3^
Cys-GCA	chr1:93981834-93981906	143.2	5.6	7.64E-08	7.96E-07
Tyr-GTA	chr2:27273650-27273738	113.6	4.0	6.20E-06	4.46E-05
Ala-TGC	chr6:28726141-28726212	189.6	3.5	1.31E-08	1.58E-07
Lys-CTT	chr16:3225692-3225764	1540.0	−3.4	4.95E-11	8.13E-10
Arg-CCG	chr17:66016013-66016085	138.3	−3.9	7.20E-06	5.00E-05
	chr6:28849165-28849237	129.4	−3.9	5.07E-09	6.33E-08
	chr15:79152904-79152976	8332.2	−4.0	5.43E-16	2.00E-14
Glu-TTC	chr13:45492062-45492133	2948.1	−4.1	8.19E-16	2.84E-14
	chr14:58706613-58706685	3533.9	−4.3	3.52E-14	1.10E-12
	chr16:3241501-3241573	7938.2	−4.5	3.12E-16	1.22E-14
	chr16:3207406-3207478	374.3	−4.6	2.34E-08	2.71 E-07
Val-TAC	chr6:27258405-27258477	582.8	−4.6	3.99E-07	3.72E-06
	chr2:131094701-131094772	612.6	−4.6	8.66E-17	3.61E-15
Val-CAC	chr5:180649395-180649467	10608.0	−5.0	7.75E-10	1.03E-08
Glu-CTC	chr6:28949976-28950047	14851.5	−5.0	6.09E-24	9.52E-22
	chr1:161431809-161431880	3246.5	−5.0	1.63E-24	4.07E-22
	chr13:41634874-41634945	591.9	−5.0	2.14E-15	7.05E-14
	chr1:161424398-161424469	3189.5	−5.0	8.06E-20	4.20E-18
	chr1:161439189-161439260	3227.3	−5.1	1.85E-23	2.27E-21
	chr19:4724647-4724719	1610.7	−5.1	1.29E-11	2.59E-10
	chr1:249168447-249168518	14777.2	−5.2	1.95E-24	4.07E-22
	chr5:180600650-180600722	39309.5	−5.3	4.09E-10	5.80E-09
	chr6:126101393-126101464	3240.3	−5.3	3.56E-23	3.18E-21
	chr6:26538282-26538354	38899.7	−5.4	1.56E-10	2.27E-09
	chr1:145399233-145399304	3225.7	−5.4	1.45E-24	4.07E-22
	chr1:161417018-161417089	3243.8	−5.4	2.18E-23	2.27E-21
	chr5:180524070-180524142	38336.8	−5.8	1.35E-10	2.00E-09
	chr5:180529253-180529325	10581.3	−5.9	8.73E-11	1.40E-09
	chr1:149298555-149298627	10125.3	−6.0	2.66E-11	4.90E-10
	chr1:149684088-149684161	10446.6	−6.1	3.98E-11	6.91E-10
	chr15:26327381-26327452	2569.5	−6.2	6.61E-22	5.16E-20
	chr1:161369490-161369562	10640.4	−6.3	4.74E-11	8.01E-10
Val-AAC	chr3:169490018-169490090	36234.5	−6.5	1.51E-11	2.96E-10
Trp-CCA	chr17:8089676-8089747	145.1	−6.6	4.53E-08	4.96E-07
	chr5:180596610-180596682	37347.2	−6.6	1.11E-11	2.31E-10
	chr5:180645270-180645342	9935.0	−6.6	1.79E-12	4.47E-11
	chr6:27648885-27648957	10105.2	−6.6	1.05E-11	2.26E-10
	chr5:180591154-180591226	37053.9	−6.7	5.06E-12	1.17E-10
Gly-CCC	chr1:17053780-17053850	151.8	−6.7	3.84E-11	6.85E-10
	chr6:27721179-27721251	10156.5	−6.8	4.99E-12	1.17E-10
	chr6:27618707-27618779	9941.2	−6.9	6.51E-12	1.45E-10
	chr11:59318102-59318174	484.7	−8.2	2.03E-13	5.76E-12
Arg-TCT	chr1:94313129-94313213	3283.4	−10.0	3.88E-18	1.73E-16
	chrX:18693029-18693101	459.1	−10.4	3.06E-19	1.47E-17
	chr6:27248049-27248121	18754.0	−11.2	1.46E-12	3.81E-11
	chr17:19411494-19411565	172.1	−12.6	9.25E-13	2.51E-11
	chr6:27173867-27173939	215.8	−14.0	3.75E-14	1.12E-12
	chr1:149294666-149294736	1407.1	−23.4	2.35E-21	1.63E-19
	chr6:27203288-27203360	655.2	−24.4	5.65E-21	3.53E-19

1 tRNA isoacceptor identity with corresponding genomic positions in the human hg19 genome. All small RNAs are derived from the 5′ end of tRNAs.

2 Average tRNA read counts-per-million computed over all libraries and taking into account the estimated dispersions and the libraries sizes. It represents a measure of the overall expression level of the tRNA fragments.

3 Fold change, P value and FDR for differential abundance were computed by EdgeR from pairwise comparisons for each tRNA fragment between the control and cancer groups.

#### YRNA-derived small RNAs

Similarly to 5′ tRNA halves, we analyzed serum levels of YRNA-derived small RNAs and found that their serum levels are altered, either increase or decrease, in HNSCC subjects (Table 4). Among the 19 types of the decreased YRNA-derived small RNAs, 11 were derived from the 3′ end, and 8 were derived from the 5′ end of YRNA genes. Only two YRNA-derived small RNAs increased in abundance, and both of them were derived from the 5′ ends of YRNA genes. We have previously observed that small RNAs derived from the 5′ end of YRNAs are more abundant in serum that those derived from the 3′ end [27, 30]. We also, have found that small RNAs derived from the 3′ end of YRNAs are 25-29 nt in size, while those derived from the 5′ end can be 25-29 nt or 30-33 nt [27]. However, in HNSCC subjects slightly more small RNAs derived from the 3′ than the 5′ end of YRNAs display altered serum levels. This observation is consistent with our previous finding that, despite the preponderance of the 5′ YRNA fragments, many more 3′ than 5′ YRNA fragments displayed altered serum levels associated with breast cancer [27]. Thus, there may be a specific association between at least these two types of cancer and the minor population of circulating small RNAs derived from the 3′ end of YRNAs. The functional significance of this specificity in size and YRNA end is not clear, especially since there is little known about the biological role of YRNA-derived small RNAs.

**Table 4:** HNSCC-associated changes in the serum levels of YRNA-derived small RNAs

YRNA^1^	YRNA end^2^	Genomic coordinates	CPM^3^	FC^4^	Pvalue^4^	FDR^4^
RNY1	5′	chr7:148684228-148684340	18001.3	4.2	2.70E-30	1.24E-20
RNY4P17	5′	chr4:169926400-169926495	198.7	2.5	1.34E-08	1.80E-08
RNY4P1	3′	chr9:77462422-77462517	4402.8	−2.1	4.57E-05	3.20E-06
RNY4P5	3′	chr8:124056957-124057052	4564.0	−2.1	3.08E-05	3.08E-06
RNY4P8	3′	chr17:38399475-38399571	4059.5	−2.2	4.93E-05	1.77E-06
RNY4P11	3′	chr20:18309650-18309746	4163.5	−2.2	3.52E-05	1.62E-06
Y_RNA.725	3′	chr9:72926523-72926617	198.5	−2.4	7.15E-08	5.96E-08
Y_RNA.122	5′	chr17:41149933-41150024	139.4	−2.5	2.54E-22	7.75E-08
RNY4P20	5′	chr6:151619976-151620068	1896.0	−2.5	3.61E-31	6.21E-09
RNY4P25	3′	chr1:151411476-151411571	407.5	−2.5	2.84E-07	9.28E-09
RNY4P27	5′	chr13:95988360-95988455	1290.9	−2.7	6.58E-29	2.75E-10
Y_RNA.182	3′	chr11:33025796-33025891	2049.6	−2.7	2.16E-07	1.59E-10
Y_RNA.257	3′	chr11:3685045-3685141	1575.3	−2.8	8.02E-08	5.43E-11
RNY4P18	3′	chr9:113859605-113859693	181.9	−2.8	2.14E-09	1.59E-10
Y_RNA.44	5′	chr22:41461558-41461650	195.9	−7.1	1.89E-82	1.42E-31
RNY4P6	5′	chr11:116886613-116886708	772.2	−7.2	8.69E-39	3.72E-35
Y_RNA.668	5′	chr11:118841208-118841305	216.4	−7.6	2.84E-85	1.79E-34
Y_RNA.7	5′	chr15:26063130-26063217	548.3	−10.8	1.45E-105	4.77E-48
Y_RNA.796	5′	chr2:201727875-201727970	536.7	−16.5	4.33E-114	1.74E-61
Y_RNA.292	3′	chr10:90345395-90345490	266.3	−100.3	1.39E-98	1.88E-106
Y_RNA.662	3′	chr2:122360649-122360740	153.7	−129.1	5.13E-90	4.09E-91

1 YRNA identity with corresponding genomic positions in the human hg19 genome.

2 Indicates whether the sequencing reads map to the 5′ or 3′ end of YRNAs.

3 Average YRNA read counts-per-million computed over all libraries and taking into account the estimated dispersions and the libraries sizes. It represents a measure of the overall expression level of the YRNA fragments.

4 Fold change, Pvalue and FDR for differential abundance were computed by EdgeR from pairwise comparisons for each YRNA fragment between the control and cancer groups.

## DISCUSSION

MicroRNAs are known to be regulators of critical steps in the pathogenesis of cancer. They have been implicated in all stages of neoplastic progression including proliferation, apoptosis, initiation, progression, invasion, chemo/radiotherapy resistance, metastasis, and relapse (reviewed in [31]). In the present study we found 7 novel and 28 known miRNAs either increasing or decreasing their circulating levels in the cancer patients as compared to normal controls. Several circulating miRNAs that we found deregulated in HNSCC patients were previously reported deregulated (often changing in the same direction) in distinct types of cancer [32-35]. Remarkably, circulating miRNA is miR-103-3p/107 was found significantly associated with the size and/or extent (reach) of the primary tumor (T). Although the number of cancer patients in our study was small, this result suggests the potential clinical utility of these miRNAs for non-invasive staging of HNSCC. Future work on independent larger cohorts is guaranteed to validate and extend these findings.

**Figure 5:**
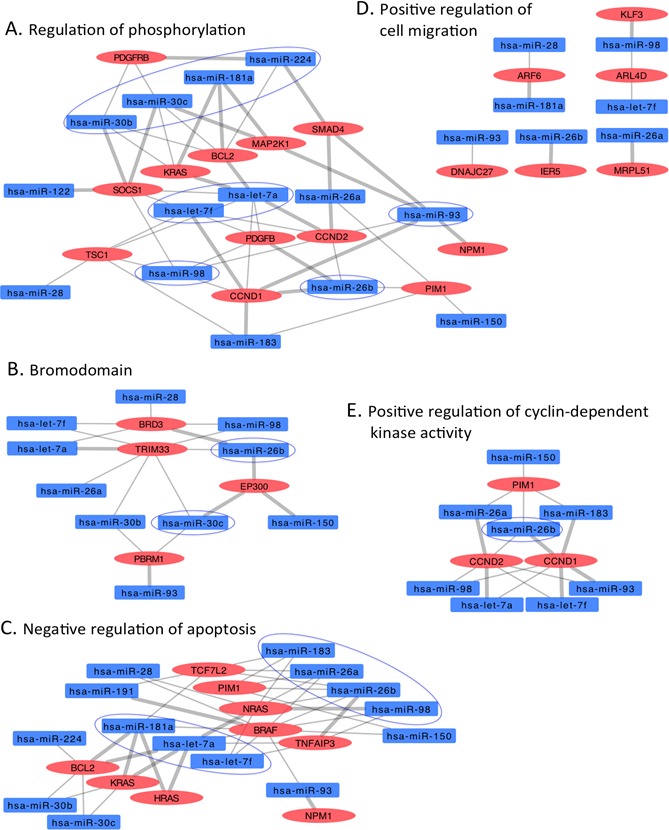
Interaction networks of COSMIC cancer consensus genes overtargeted by downregulated circulating HNSCC miRNAs Subnetworks represent functional categories significantly enriched in sets of genes overtargeted by downregulated miRNAs. **A.** regulation of phosphorylation, **B.** bromodomain, **C.** negative regulation of apoptosis, **D.** positive regulation of cyclin-dependent kinase activity. Thick grey edges highlight validated miRNA-mRNA interactions, thin edges represent predicted interactions, ovals represent COSMIC genes overtargeted by downregulated HNSCC miRNAs (rectangles); color-coded red indicates upregulation, blue indicates downregulation. Circled miRNAs putatively target three or more genes in the specific subnetwork.

The circulating DA miRNAs in our HNSCC patients appear to coordinately and non-randomly target a group of genes relevant to cancer development and progression. These genes, identified by selecting DA miRNA-overtargeted genes (which interact with more DA miRNAs than expected by chance) that are found mutated in cancer tissue (as reported by the COSMIC database census [36]), are enriched in biological functions such as regulation of apoptosis, cell cycle, blood vessel morphogenesis (angiogenesis), immune system activation, and transcription among others. As evidenced in Figures 4 and 5, the interaction network approach underscores the key role of let-7a/f, miR-26a/b, miR-103, miR-107, miR-205, and miR-320a/b among others. Supporting our findings, multiple miRNA-mRNA interactions identified by our analysis have been experimentally validated (thick grey edges in the networks) [37] and several DA circulating miRNAs found deregulated in distinct types of tumor tissues including HNSCC [38-44].

Chen and colleagues showed that miR-103 and miR-107 target the metastasis suppressors DAPK and KLF4 [45]. Notably, our over-representation analysis demonstrated that DAPK and KLF4 were overtargeted by circulating miRNAs increasing expression in the HNSCC serum samples. Importantly, according to published data, the DAPK gene is one of the more often methylated in laryngeal squamous cell carcinoma [46] and ablation of Klf4 in mice results in more severe dysplastic lesions in oral mucosa and higher incidence of squamous cell carcinoma [47]. Another *in vitro* study demonstrated the interaction of miR-103 with GPRC5A, a G-protein-coupled receptor that has been associated with a variety of cancers (reviewed in [48]). Other authors demonstrated that miR-103 is an oncomiR that promotes colorectal cancer proliferation and migration through down-regulation of the tumor suppressor genes DICER and PTEN [49]. Relevantly, our interaction network analysis of miRNA-overtargeted genes underscored the central role of these interactions (Figure 4). Moreover, NF1 (a negative regulator of the ras signal transduction pathway) is also overtargeted by miR-103/107 and two other HNSCC upregulated miRNAs. Loss of NF1 expression in human endothelial cells promotes autonomous proliferation in human endothelial cells [50]. Furthermore, high expression of miR-103/107 was found correlated with poor survival in human esophageal cancer [41].

In contrast to the clear oncogenic role demonstrated for miR-103a and miR-107, miR-320 has been mainly reported as an anti-angiogenic miRNA in breast cancer [51] and oral squamous cell carcinoma [43]. This may indicate that miR-320 overexpression in the circulation of our HNSCC patients is part of an adaptive response against the cancer. Alternatively, miR-320 might perform as an oncomir under certain conditions (e.g., distinct miRNA partners involved in cooperative gene targeting could inhibit the activity of distinct subsets of genes). The report by Kim and Choi [52] supports the oncogenic potential of miR-320. These authors found that miR-320 promotes proliferation of Dgcr8-deficient embryonic stem cells (ESCs) by releasing them from G1 arrest. This is accomplished by inhibition of the cell cycle inhibitors p57 and p21. Notably, our interaction network analysis of miRNA-overtargeted genes showed that miR-320 is involved in several interactions that target important tumor suppressor genes such as CDKN2A and PTEN (Figure 4). Mutations in the CDKN2A gene are found in approximately 25% of head and neck squamous cell carcinomas (HNSCC). Most of these mutations lead to production of little or no functional p16(INK4a), a protein that regulate cell growth and division (reviewed in [53]). Recently, low PTEN expression was found associated with worse overall survival in head and neck squamous cell carcinoma patients treated with chemotherapy and cetuximab [54].

In addition, we detected increased levels of miR-205 in the circulation of HNSCC patients as compared to healthy subjects. Importantly, we also found increased expression of miR-205 in HNSCC tumor tissue as compared to healthy tissue from the same patients in a different cohort (unpublished data). Supporting our findings, Tsukamoto and colleagues found correlation between the changes in the expression of a group of endometroid endometrial carcinoma (EEC)-associated miRNAs (including miR-205) in both tissue and plasma [42]. These authors proposed that these miRNAs represent potential non-invasive biomarkers for EEC. Similarly, the relative expression of miR-205-5p was significantly higher in both non-small cell lung carcinoma tissue (compared with cancer-adjacent paired specimens) and serum (compared with serum from benign pulmonary condition patients and healthy volunteers) [55]. This miRNA was also found upregulated in high-grade serous ovarian carcinoma [56]. Moreover, miR-205-5p and members of the miR-200 family target epithelial-mesenchymal transition regulators (ZEB1 and SIP1), apparently being important in tumor progression [57].

Regarding to miRNAs with reduced levels in the circulation of the HNSCC patients, our interaction network analysis underscores the key role of let-7a/f and miR-26a/b, among others. As highlighted in Figure 3, several of these interactions (i.e., with BCL2, BRAF, BRD3, CCND1, CCND2, EP300, FOXA1, HRAS, KRAS, NRAS, MAP2K1, NPM1, PBRM1, PDGFRB, SMAD4, SOCS1) have been previously validated [37]. Our results suggest that depletion of these miRNAs in the circulation of HNSCC patients will allow overexpression of the respective target genes in specific target tissues. Indeed, we demonstrate the overtargeting of important oncogenes by these miRNAs. Adding support, let-7 has been shown to function as a tumor suppressor by regulating multiple oncogenic signaling pathways, therefore, its downregulation would promote cancer. Yang and colleagues demonstrated that a polymorphism in the let-7 binding site in the 3′-UTR of the KRAS gene was associated with increased oncogenic KRAS expression in an *in vitro* model [58]. Furthermore, this polymorphism was found associated with increased cancer risk in non-small-cell lung cancer patients and reduced overall survival in oral cancers [38, 58], suggesting functional and clinical significance. In addition, Yu and colleagues demonstrated that let-7a expression was significantly reduced in head and neck cancer (HNC) tissues as compared to adjacent normal cells. These authors showed that let-7a negatively modulates the expression of stemness genes and plays a role as a tumor suppressor in HNC [59]. Our analysis revealed that let-7a and let-7f are overtargeting important cell cycle genes with demonstrated roles in HNC and other types of cancers (Figure 4) [60-62]. In particular, the expression of CCDN1 was found to be regulated by LIN28A via the inhibition of let-7 miRNA biogenesis in cancer cells [61]. Similarly, direct regulation of CCND2 via a let-7/Lin28b mechanism was also validated in an HNC cell line [60].

MicroRNAs miR-26a and miR-26b have also been found downregulated in distinct types of cancer tissue, including squamous cell carcinoma of the tongue [63]. Recently, Lu and colleagues reported that miR-26a is commonly downregulated in nasopharyngeal carcinoma (NPC) and functions by repressing EZH2 expression [44]. In addition, the *in vitro* expression of miR-26 in liver cancer cells induced cell-cycle arrest associated with direct targeting of cyclin D2 [64]. Similarly, our interaction network analysis revealed that cyclin D2 is overtargeted by HNSCC DA miRNAs including miR-26a/b. Remarkably, Kota and colleagues found that systemic administration of miR-26a in a mouse model of hepatocellular carcinoma resulted in inhibition of cancer cell proliferation, induction of tumor-specific apoptosis, and dramatic protection from disease progression [64].

Family members of the miR-183 are proposed as promising biomarkers for early cancer detection, prognosis, and as therapeutic targets in several cancers [65-67]. However, the results of their expression profiling in cancer tissues are inconsistent and controversial (Reviewed in [68]). Members of this miRNA family has been mainly reported as oncomirs that inhibit apoptosis and promote proliferation and invasion in different types of carcinomas [69, 70]. Our work provides evidence for a potential tumor suppressor role of miR-183, which we found depleted in HNSCC serum samples. Another controversial miRNA is miR-93, depleted in the serum of HNSCC patients. MicroRNA miR-93, has been found deregulated in head and neck cancer and often reported as an oncomir [71, 72]. However, our results and those of other investigators suggest a tumor suppressor role for this miRNA [73].

Our analysis showed that miR-150 (also reduced in the serum of the HNSCC patients) appears to target several genes involved in the regulation of cell growth and division such as PIM1 and EP300 (Figure 4). Particularly, the interaction of miR-150 with EP300 was previously reported in the regulation of high glucose-induced cardiomyocyte hypertrophy [74]. This miRNA functions as a tumor suppressor in different types of human cancers [2, 75, 76]. Regarding to HNSCC, Persson and colleagues described a new mechanism of activation of the oncogene MYB in human HNC by deleting conserved target sites for miR-150 [2]. In addition, a full exome and transcriptome sequencing of a large set of HNSCC-derived cells revealed that most HNSCC cells harbor multiple mutations and copy number variations in the 3′-UTR of EP300 that encompases the miR-150 binding site may contribute to HNSCC initiation and progression [77]. Moreover, the expression of miR-98 (depleted in our HNSCC samples) was significantly lower in esophageal squamous cell carcinoma tissues as compared to matched normal tissues [78], and in human samples from squamous cell carcinomas of the oral cavity [79]. This study highlighted the role of miR-98 in the regulation of tumor metastasis by inhibiting migration and invasion in a cell line of esophageal squamous cell carcinoma [78].

Remarkably, most of the genes overtargeted by DA miRNAs and playing central roles in our HNSCC miRNA-mRNA interaction networks (such as DICER, PTEN, CDKN2A, NOTCH, MDM2, CCND1, HRAS, and SMAD4 among others) (Figures 4 & 5) were previously reported within a group of genes altered in HNSCC (reviewed in [9]). Moreover, a comprehensive integrative genomic study of HNSCC just reported by The Cancer Genome Atlas Network showed that several of these genes (i.e., HRAS, PTEN, NOTCH, SMAD4 and CDKN2A and CCND1) are more often altered in HPV(−) tumors [8].

By conducting miRNA-mRNA interaction network analysis of overtargeted COSMIC genes (triple-filtering strategy described in Material and Methods), we demonstrated that the same genes/pathways affected by somatic mutations in cancer are affected by changes in the abundance of miRNAs that target the respective relevant genes. Therefore, one important conclusion from our work is that during cancer development, there seems to be a convergence of oncogenic processes driven by somatic mutations and/or miRNA regulation affecting key cellular pathways.

In addition to miRNAs, we detected changes in serum abundance of two other major classes of circulating sncRNAs (i.e., tRNA- and YRNA-derived small RNAs) in HNSCC patients when compared to healthy donors with no known pathologies. Despite the small number of cases used in this study, the multi-dimensional scaling analysis of miRNA, and tRNA- and YRNA-derived small RNAs showed a significant separation between cancer and normal samples indicating low biological coefficient of variation (Figure 2). This result also reflects a strong association between the cancer diagnosis and the changes in the circulating levels of the small RNAs, which suggests important mechanistic role in cancer biology (Figure 2). Consistent with this idea, some of the functions attributed so far to tRNA- and YRNA-derived small RNAs are involved in carcinogenesis. Recently, tRNA-derived small RNAs [80] and full length tRNAs [81-86] have been shown to inhibit apoptosis by sequestering cytochrome c. Also, blocking the formation of tRNA-derived small RNAs by inhibiting tRNA cleavage slows tumor development [87]. The tRNA-derived stress-induced small RNAs, which suppress global protein translation, seem to reprogram protein translation in response to stress, thereby promoting cell survival when cells are exposed to unfavorable conditions [88-90]. Promotion of injured cells survival instead of apoptosis allows damaged cells to persist and acquire abnormalities that alter tissue microenvironment and promote cancer. YRNAs or their derivatives are linked to processes relevant to DNA replication and cell proliferation [91-93], stress responses [94], apoptosis [13], viral infections [95, 96], senescence [97-99], and several types of cancer [100, 101]. We have recently found that changes in serum levels of specific types of both YRNA- and tRNA-derived small RNAs are associated with breast cancer and its clinicopathological characteristics [27]. The association between cancer and changes in these novel circulating RNA species raises the possibility of a causal connection between carcinogenesis and the tRNA- and YRNA-derived small RNAs. Even though it is tempting to speculate that tRNA- and YRNA-derived small RNAs may mediate systemic effects of cancer, it remains to determine whether they originate from the cancer itself, or from peripheral cells affected by the cancer. Does cancer itself change the serum composition of tRNA- and YRNA-derived small RNAs, or is the change caused by some physiological response to the cancer? A more definite answer requires elucidation of the secretion pathways of extracellular YRNA- and tRNA-derived small RNAs, the cells that produce and release them in the extracellular environment, their packaging inside cells, their transport and delivery to their destination, and the functions they may exert once inside recipient cells to contribute to malignancy.

The presence of tRNA- and YRNA-derived small RNAs in the bloodstream, in addition to miRNAs, and the alterations of their levels in cancer patients provide strong foundation for exploring these circulating RNA species as new noninvasive biomarkers. This may lead to the development of a multi-marker signature optimal for early detection of cancer. A signature with more than one type of markers (for instance, tRNA- and YRNA-derived small RNAs in addition to miRNAs) would be more successful in clinical use because it is likely less sensitive to biological differences than signatures with miRNAs only. Establishing a reliable signature of circulating sncRNAs in HNSCC will allow for early detection, prediction of prognosis, and more precise adjustment of the therapeutic approach for each individual patient, therefore reducing the complications related to under- or over-treatment of the patients affected by HNSCC.

## MATERIALS AND METHODS

### Blood collection and serum preparation

Serum was prepared in the Department of Head and Neck Surgery, Greater Poland Cancer Center before surgical treatment from 7 males (Mean age = 56.3 years and STDEV = 7.6) diagnosed with HNSCC (Table 1) and in the health clinic at the University of California-Riverside from 7 healthy control males (Mean age = 65.4 years and STDEV = 2.7). The Institutional Review Board of University of Medical Sciences in Poznan approved the study, and informed consents were obtained from all patients. The University of California-Riverside Institutional Review Board approved the collection of blood from volunteers who served as the control group in this study. Blood samples were collected in BD Vacutainer Serum Separation Tubes, incubated for 15 minutes at room temperature to allow coagulation, and centrifuged at 1300 g for 10 minutes. The serum supernatant was transferred to new tubes, centrifuged at 16,000 g for 15 minutes to remove any residual cells and debris, and stored at −80°C.

### Exclusion criteria

Following the study protocol, patients with local recurrences, second primary tumor and HPV positive were excluded from experimental groups. Patients with a previous history of chemo- or radiotherapy were also excluded.

### RNA isolation and small RNA library construction

Total RNA, including small RNA, was isolated using the miRNeasy kit (Qiagen) from 0.4 mL of serum from each participant. RNA isolated from each serum sample was used to construct sequencing libraries with the Illumina TruSeq Small RNA Sample Prep Kit (Illumina). Briefly, 3′ and 5′ adapters were sequentially ligated to small RNA molecules and the obtained ligation products were subjected to a reverse transcription reaction to create single stranded cDNA. To selectively enrich fragments with adapter molecules on both ends, the cDNA was amplified with 15 PCR cycles using a common primer and a primer containing an index tag to allow sample multiplexing. The amplified cDNA constructs were gel purified, and validated by checking the size, purity, and concentration of the amplicons on the Agilent Bioanalyzer High Sensitivity DNA chip (#5067-4626, Genomics Agilent, Santa Clara, CA). The libraries were pooled in equimolar amounts, and sequenced on an Illumina HiSeq 2000 instrument to generate 50-base reads.

### Bioinformatics and statistics analyses of circulating small RNA sequencing reads

#### a Alignment and annotation of sequencing reads

Sequencing reads were pre-processed and mapped to the human (hg19) genome with Bowtie version 0.12.8 [102] using the “end-to-end k-difference (−v)” alignment mode and allowing up to 2 mismatches. In addition, this mode of alignment was combined with options (−k 1 –best) that instructed Bowtie to report only the best alignment if more than one valid alignment exists. Annotation of the mapped sequencing reads was performed with BEDTools [103] using non-coding RNAs from Ensembl GRCh37 release 70, miRNAs from miRBase 20 (www.mirbase.org), and tRNAs from Genomic tRNA Database [104].

#### b Generation of the expression values of circulating tRNA- and YRNA-derived small RNAs

The Bowtie alignment files described above were analyzed with BEDTools [103] to count the reads that align to tRNA genes in the Genomic tRNA Database [104] and YRNA genes and pseudogenes in the noncoding RNAs of Ensembl GRCh37 release 70.

#### c Detection of miRNAs and generation of their expression values with miRDeep2

Sequencing reads were analyzed with miRDeep2 [28], a probabilistic algorithm based on the miRNA biogenesis model and designed to detect miRNAs from deep sequencing reads. Briefly, miRDeep2 removes the 3′ adapter sequence and discards reads shorter than 18 nucleotides, before aligning them to the human hg19 genome. Only reads that map perfectly to the genome five or less times are used for miRNA detection, since human miRNAs usually map to few genomic locations. For the purpose of analyzing the sequenced miRNAs, the known miRNA input was from miRBase v.20 [105], and Mus musculus was designated as the related species. The miRDeep2 algorithm uses a miRNA biogenesis model to detect known miRNAs and discover novels miRNAs. It aligns reads to potential hairpin structures in a manner consistent with Dicer processing and assigns scores that measure the probability that hairpins are true miRNA precursors; it also estimates the expression levels of the identified miRNAs.

#### d Differential expression analysis of the circulating small RNAs

Expression values of miRNAs and tRNA- and YRNA-derived small RNAs obtained as described above were analyzed with the Bioconductor package edgeR [29] to detect statistical differences in the levels of circulating small RNAs between HNSCC and control groups. The algorithm of edgeR uses the negative binomial model to measure differential gene expression. Expression values were normalized for the libraries size with the trimmed mean of M-values (TMM) method. The overall degree of inter-library variability in the data and the coefficient of biological variation (BCV) were measured by estimating first the common then the tagwise dispersions as recommended for experiments with a single factor. The differential expression is determined by the exact test which is only applicable to experiments with a single factor. P-values were adjusted for multiple testing using the Benjamini and Hochberg method to control the false discovery rate (FDR). Differences in expression were considered significant below an FDR of 15%.

#### e Correlation analysis

Circulating miRNA levels were tested for normal distribution using the Shapiro-Wilk Normality Test and subjected to correlation analysis with tumor staging. Pearson correlation for normally distributed data and Nonparametric Spearman correlation for non-normal data) in GraphPad Prism 6.05.

#### f Prediction of peripheral genes potentially targeted by differentially abundant circulating miRNAs

The databases miRBase [105] and TargetScan [106] from the R packages microRNA [107] targetscan.Hs.eg.db [108] were used to predict the genes that are potentially targeted by the differentially abundant miRNAs. Lists of putative targets are generated by the intersection of the targets predicted from TargetScan and miRBase.

#### g Over-representation analysis of miRNA-targeting events on predicted targets and miRNA-mRNA interaction network construction

Statistical hypergeometric tests were implemented in the R environment for comparison of observed proportions of differentially abundant (DA) miRNA interaction events on each predicted target mRNA versus expected proportions in the predicted miRNA-mRNA interaction universe. Validated interactions from miRTarBase 4.5 were added to the interaction universe. The overtargeted genes (*P* < 0.01, FDR < 0.05) were then additionally filtered by selecting those included in the list of cancer genes reported by the COSMIC database (genes containing somatic mutations in cancer tissue) [36] and analyzed with the Functional Annotation Clustering tool from the DAVID Bioinformatics Database [109] to determine which pathways and GO categories are enriched in the gene list. The subset of overtargeted COSMIC genes that are found functionally enriched were then used to filter the list of interactions between DA miRNAs and putative overtargeted mRNAs. The subset of relevant interactions was then used to build miRNA-mRNA interaction networks using the Cytoscape 3.0.2 software [110].

## SUPPLEMENTARY FIGURES AND TABLES








