# Supplementary material for: Circulating small non coding RNA signature in head and neck squamous cell carcinoma
Source: Oncotarget. 2015 May 25;6(22):19246–63. doi: 10.18632/oncotarget.4266 (PMC4662488; doi:10.18632/oncotarget.4266)
Supplement: Supplementary file 1 [file oncotarget-06-19246-s001.pdf]

Circulating small non coding RNA signature in head and neck squamous cell carcinoma

Supplementary Material

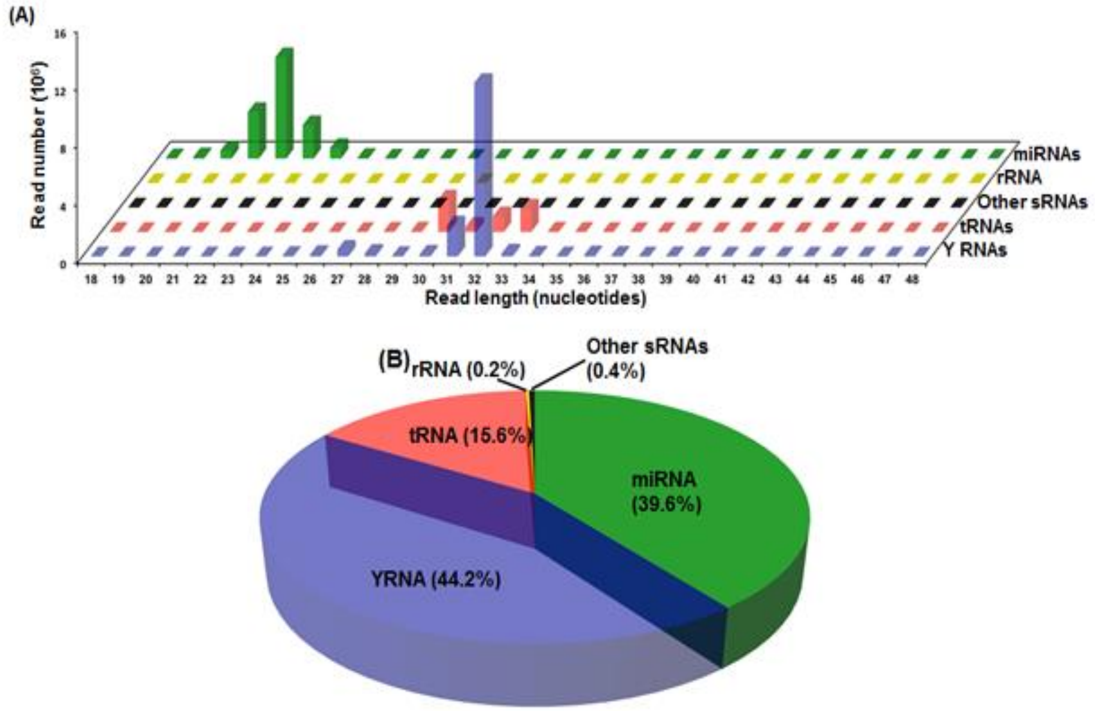

Supplementary Figure S1: Length distribution and annotation of sequencing reads from normal serum small RNAs. Sequencing reads from normal samples were pooled to analyze the quality of reads, i.e., length distribution, and the types and proportions of small RNAs from which the reads are derived from. Pooling is used only to examine the general characteristics of the reads, and not to measure the differential expression of small RNAs between control and cancer groups. A: Plot of length against abundance of pooled mapped reads according to their annotation as miRNAs, YRNAs, tRNAs, rRNAs, or other sRNAs (snRNAs and snoRNAs). B: Pie chart showing the percent of reads mapping to the indicated types of small RNAs in pooled datasets obtained by sequencing of small RNAs in normal serum.

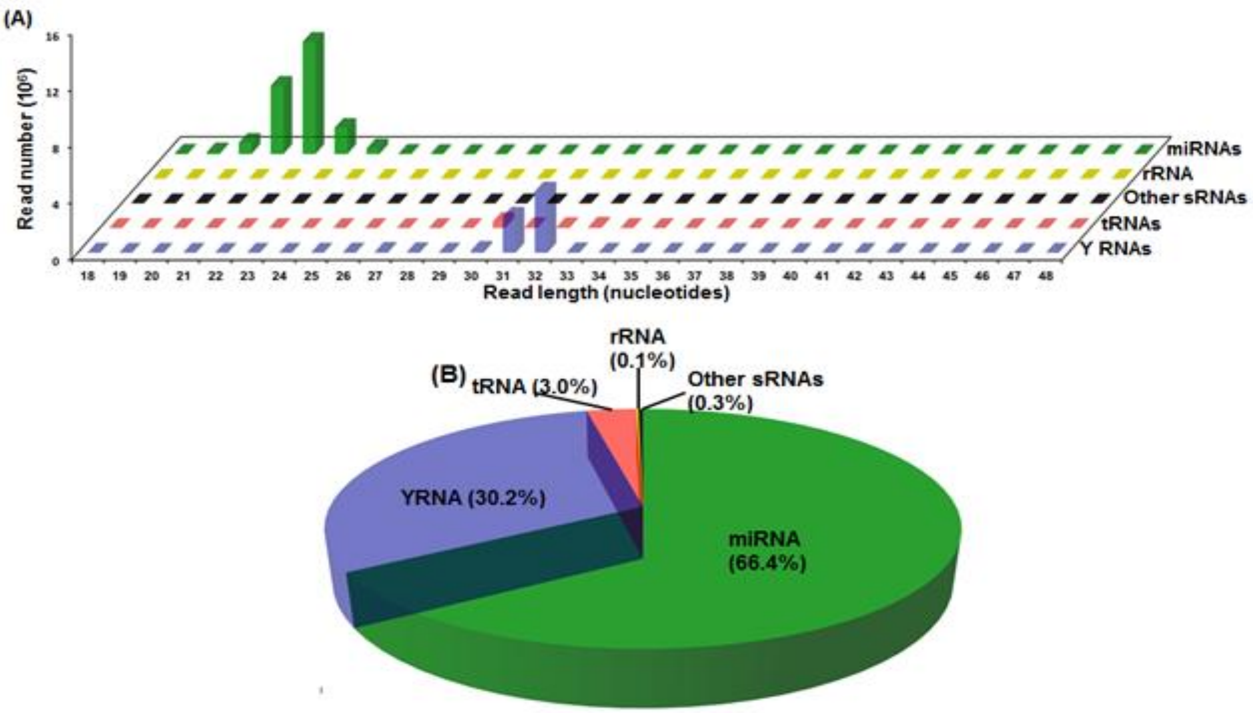

Supplementary Figure S2. Length distribution and annotation of sequencing reads from cancer serum small RNAs. Sequencing reads from normal samples were pooled to analyze the quality of reads, i.e., length distribution, and the types and proportions of small RNAs from which the reads are derived from. Pooling is used only to examine the general characteristics of the reads, and not to measure the differential expression of small RNAs between control and cancer groups. A: Plot of length against abundance of pooled mapped reads according to their annotation as miRNAs, YRNAs, tRNAs, rRNAs, or other sRNAs (snRNAs and snoRNAs). B: Pie chart showing the percent of reads mapping to the indicated types of small RNAs in pooled datasets obtained by sequencing of small RNAs in normal serum.

Supplementary Table S1. Venn diagram of HNSCC overtargeted genes and COSMIC genes.

Supplementary Table S2. DAVID Functional Annotation Clustering of 48 COSMIC genes overtargeted by upregulated HNSCC miRNAs.

Supplementary Table S3. DAVID Functional Annotation Clustering of 76 COSMIC genes overtargeted by downregulated HNSCC miRNAs.
